# Trends in prevalence of substance use among Icelandic adolescents, 1995–2006

**DOI:** 10.1186/1747-597X-3-12

**Published:** 2008-05-28

**Authors:** Inga D Sigfusdottir, Alfgeir L Kristjansson, Thorolfur Thorlindsson, John P Allegrante

**Affiliations:** 1 Icelandic Centre for Social Research and Analysis, School of Health and Education, Reykjavik University, Ofanleiti 2, 103 Reykjavik, Iceland; 2 Department of Sociology, Faculty of Social Science, University of Iceland, Oddi by Sturlugata, 101 Reykjavik Iceland, and Institute for Public Health, Laugarvegur 116, 105 Reykjavik, Iceland; 3 Department of Health and Behavior Studies, Teachers College, 525 W 120th Street, Columbia University, New York, NY 10027, USA; 4 Department of Sociomedical Sciences, Mailman School of Public Health, Columbia University, 722 West 168th Street, New York, NY 10032, USA

## Abstract

**Background:**

Adolescent substance use continues to be of great global public health concern in many countries with advanced economies. Previous research has shown that substance use among 15–16 year-old-youth has increased in many European countries in recent years. The aim of this study was to examine trends in prevalence of daily smoking, alcohol intoxication, and illicit substance use among Icelandic adolescents.

**Methods:**

Repeated-measures, population-based cross-sectional surveys of between 3,100 and 3,900 10th-grade students who participated in the annual *Youth of Iceland *studies were analyzed, with response rates of between 80% and 90%.

**Results:**

The prevalence of daily smoking, alcohol intoxication, and illicit substance use was at a peak in 1998, with almost 23% having reported daily smoking, 42% having reported becoming intoxicated at least once during the last 30 days, and over 17% having used hashish once or more often in their lifetime. By 2006, daily smoking had declined to 12%, having become intoxicated once or more often during the last 30 days to 25%, and having ever used hashish declined to 9%.

**Conclusion:**

The prevalence of substance use among Icelandic 10^th ^graders declined substantially from 1995 to 2006. Proportions of adolescents who smoke cigarettes, had become intoxicated during the last 30 days, as well as those admitting to hashish use all decreased to a great deal during the period under study. The decline in prevalence of adolescent substance use in Iceland is plausibly the result of local community collaboration where researchers, policy makers and practitioners who work with young people have combined their efforts.

## Background

Nationally representative surveys, conducted in several European countries as well as in the US and Australia, have become essential for monitoring drug use among adolescents [1-3]. Such surveys have increasingly been used as a basis for policy-making, including the assessment of risk factors for drug use and in the evaluation of programs designed to reduce drug use. Surveys monitoring drug use and related risk factors among youth have been conducted in Iceland since the early 1980s to provide information for policy-makers pertaining to youth. In the beginning, these surveys were conducted in cooperation with the municipalities that were, in practice, responsible for the formal organization of youth activities. In 1989, the surveys became nationally representative and better connected to the international scene in youth research. Major methodological changes were introduced in 1992 when these studies became population-based instead of being based on samples. This change offered the possibility to analyze youth issues on both a national and local level, strengthening the links between policy, research and practice.

The results from the Icelandic surveys indicate that substance use among adolescents in Iceland rose gradually, but steadily, during the 1990s. The prevalence of 15- and 16-year-old students in the 10^th ^grade of school who reported, for example, that they had smoked cigarettes on a daily basis increased from 15% to 23% from 1992 to 1998, and the prevalence of those admitting to having ever used hashish in their lifetimes rose from 7% to 17% [1]. This trend paralleled the increase in substance use among 15- to 16-year-old adolescents during the 1990s and the first years of the 21st century that has been documented in many European countries and in North America [2,3].

The increase in substance use in Iceland was well documented in the national survey results. The findings, widely discussed in the Icelandic media, were alarming to the public. Apart from concern about the long-term consequences of smoking and other substance use on long-term health status, the short-term consequences of substance use, such as consumption of alcohol on automobile injuries and fatalities, and the use of amphetamines as an illegal substance, were of equal urgency. The public discussion led to a growing concern about the general well-being of youth in Iceland and a political consensus that municipalities, schools, and the national government needed to take action to do more to prevent substance use.

In response, a governmental program was developed in 1998 to stem the trend. It was initiated under the label "Drug-Free Iceland". This initiative consisted of a five-year program, led by the City of Reykjavik and the Ministry of Justice in the central Icelandic Government. The program's main goal was to commit significant national resources in support of a coordinated effort to achieve a Drug-Free Iceland. This program operated in collaboration with the National Counsel for Alcohol and Drug Prevention, which is an entity that was established by the Icelandic Government and was supervised by the Ministry of Health. Thus, the two governmental bodies worked closely together to form a coordinated prevention effort whose implementation has been unfolding between 1998 and the present.

The results from the Icelandic national surveys were used to develop an effective prevention approach with a broad-scale and systematic assessment of the risk and the protective factors that predicted adolescent substance use in Iceland. The key components of this prevention approach included:

• Educating parents about the importance of emotional support, reasonable monitoring, and increasing the time they spend with their adolescent children.

• Encouraging youth to participate in organized recreational and extracurricular activities and sports.

• Working with local schools in order to strengthen the supportive network between relevant agencies in the local community.

The research underlined the importance of the adolescent-parent relationship, the powerful influence of the peer group, and a commitment to facilitate the participation of adolescents in guided recreational and extracurricular activities, such as sports and organized youth work. The research helped to conceptualize the prevention effort as one that sought both to reduce the potentially-modifiable risk factors for substance use while at the same time strengthening community-level protective factors. Thus, the approach focused not only on reducing risk factors, but also on mobilizing society to foster responsible guardianship, community attachment, and informal social control, all on the local community level. This effort has come to be known as the *Icelandic Model of Adolescent Substance Use Prevention*. It is important to demonstrate that this approach is not merely a "program" in the conventional sense with a given time frame, but rather a long-term effort to alter society on behalf of young people in Iceland in order to decrease the likelihood of adolescent substance abuse (a paper describing the *Icelandic Model *in more detail is currently in submission).

The theoretical principles underlying this approach have been well-documented in numerous studies [1,4-14]. The cumulative research experience has provided a framework for a host of programs that may vary somewhat between local communities and municipalities, but underline the complex relationship that exists between theory, practice, and policy-making in the prevention of substance use. This work has shown that adolescents who are strongly attached to their parents and family and take part in organized youth work, such as recreational activities or sports, are significantly less likely to be involved in harmful and destructive behaviors within their peer networks. This has important implications for both intervention and policy that have been both documented in the substance-use literature in Iceland and operationalized in national substance-use prevention policy [1,15].

This paper reports the results of a study whose aim was to analyze trends in prevalence of substance use among annual groups of Icelandic 10^th ^grade students, from 1995 to 2006, using population-based data.

## Methods

### Setting

Iceland stands at a crossroad between North America and Europe and the other Nordic countries. Reykjavik, its largest city, is located in the south western part of the island, with 150,000 of the estimated 300,000 inhabitants of Iceland residing there and in its surrounding metropolitan area. Approximately 94% of Icelanders are of Norwegian and Irish-Celtic decent and 87% of the population belongs to the Lutheran State Church [16]. Although the population can be characterized as homogeneous, Iceland's popular culture is heterogeneous and is both influential in and influenced by western European and North-American social trends. Moreover, Iceland is beginning to experience some of the effects of global migration and a growing immigrant population that comes from many regions of the world.

### The Youth in Iceland Survey Data

This study utilized population-level data from *Youth in Iceland*, a national annual survey of Icelandic adolescents. This series of cross-sectional social surveys is designed to capture data on the lifestyles and the social well-being of young people in Iceland. In addition, these surveys have been utilized to monitor the impact of efforts to prevent substance use nationwide. The data collection is guided by a strict methodological protocol developed by the Icelandic Centre for Social Research and Analysis (ICSRA) at the Reykjavik University School of Health and Education. All aspects of data collection are approved by an Icelandic central human subjects review committee, use passive parental informed consent, and are supervised by the ICSRA.

Annually, since 1997, in March of each year, the *Youth in Iceland *surveys are conducted among 9^th ^and 10^th ^graders in all secondary schools in Iceland. In an island setting and with only 300,000 inhabitants, it is relatively easy to access the entire population of school children. The data collection is carried out in cooperation with the Icelandic Ministry of Education, Science, and Culture, the municipalities around the country, and an overwhelming majority of the schools. This has resulted in a set of data from the *Youth in Iceland *surveys that includes between 80% and 90% of all individuals in the age-groups studied in each year. A typical age-group includes between 4,000 and 4,500 individuals and the surveys capture data from between 3,100 and 3,900 respondents each year. Table 1 shows the number of respondents and the proportion of population they represent for each year of the *Youth in Iceland *surveys.

**Table 1:** Number of participants in the Youth in Iceland surveys, 1995–2006.

*Year*	*N*	*% of population*	*% males*
1995	3,814	86	51
1997	3,912	90	51
1998	3,723	89	52
1999	3,549	87	50
2000	3,220	82	49
2001	3,069	79	47
2002	3,226	78	50
2003	1,699	37	52
2004	3,805	85	52
2005	3,713	78	50
2006	3,670	82	50

*Note*. In 2003 the Icelandic survey became the local part of the international ESPAD study. This study used a questionnaire too large to implement in full size in class-room setting among 9^th ^and 10^th ^graders. The survey was divided into two parts (A and B), using separate questionnaires. Participants were randomly selected for these two groups. Both questionnaires included measures on substance use but only part A had items referring to illegal use such as hashish and amphetamines. The A part is utilized in this analysis.

One of the specific aims of the *Youth in Iceland *surveys is to document the current status and rates of adolescent substance use in the same age-group (not birth-cohort) annually. The study questionnaires include the identical set of questions about substance use every year. Moreover, every three years the data collection is more comprehensive; the questionnaires then include items about social circumstances and well documented risk and protective factors associated with substance use. This is done in order to evaluate whether and how emerging trends in the adolescent social environment are influencing adolescent substance use. The main categories of variables, along with background factors and rates of substance use, include: relationship with parents and family, relationship with friends and peer group influences, emotional well-being and physical shape, participation in sports and organized youth work, school attachment, and deviant behavior.

### Procedure

All aspects of this data collection were supervised by ICSRA. Teachers at individual school sites supervise the participation of the students in the study and administer the survey questionnaire guided by strict methodological instructions from the ICSRA. All students who attend school on the day that the questionnaires are scheduled to be administered complete the questionnaires inside their classrooms. Students are instructed not to write their names or social security numbers, or any other identifying information, anywhere on the questionnaires. They are instructed to complete the entire questionnaires, but to ask for help if they have any problems or any questions for clarification. Once students complete the questionnaires, they are asked to place their completed questionnaire in the envelope provided and seal it before returning the questionnaire to the supervising teacher. A prior study on our data collection methodology revealed no specific teacher effect on the responses [17].

### Measurements

We examined daily smoking, having become intoxicated during the last 30 days, having ever used hashish, and having ever used amphetamines. Daily smoking was assessed with the question, "How much on average have you smoked during the last 30 days?" (1 = "Nothing", 2 = "Less than one cigarette per week", 3 = "Less than one cigarette per day", 4 = "1–5 cigarettes per day", 5 = "6–10 cigarettes per day", 6 = "11–20 cigarettes per day", and 7 = "More than 20 cigarettes per day"). In analyzing the trends in smoking we carry out two separate analyses; first using the continuous variable described above, and second, with a collapsed measure in a dichotomized variable (0 = "Nothing or less than daily" and 1 = "Daily").

Alcohol intoxication during the last 30 days was assessed with the question, "How often have you become intoxicated during last 30 days?" (1 = "Never", 2 = "1–2 times", 3 = "3–5 times", 4 = "6–9 times", 5 = "10–19 times", 6 = "20–39 times" and 7 = 40 times or more"). In analyzing the trends in intoxication we carry out two separate analyses; first using the continuous variable described above, and second, with a collapsed measure in a dichotomized variable (0 = "No" and 1 = "Yes, once or more often").

Use of illicit substances included use of hashish (cannabis) and amphetamines. Use of hashish was assessed with the question, "How often, if ever, have you used hashish in your lifetime?". To assess how often, if ever, the respondents had used amphetamines in their lives, we asked the question, "How often, if ever, have you used amphetamines in your lifetime?". Answer categories are the same as with alcohol consumption. For both questions we carry out two separate analyses; first using the continuous variables described above, and second, with a collapsed measure in a dichotomized variable (0 = "Never" and 1 = "Yes, at some point").

### Statistical Analyses

In evaluating the prevalence of daily smoking, intoxication during last 30 days, and lifetime use of hashish and amphetamines among 10^th ^graders from 1995 to 2006, we conducted two separate analyses. First, we conducted a linear trend analysis using Analysis of Variance (ANOVA) [18]. Since the time trends in substance use might be considered quadratic, or curve-linear, in shape rather than straight-linear, we also performed a quadratic trend analysis with ANOVA. Both are reported in the results. For both the linear and the quadratic trend analyses we have transformed the responses in the dependent variables with natural logarithm because of the normality distribution assumptions in using ANOVA [19]. As a result, the span in the responses of the substance use variables decreases from 1–7 to 0–1.95. Second, because the response distribution in the substance-use variables might still be considered positively biased, even after the natural logarithm transformation of the responses (because most people claim they have never used these substances), we have also calculated the odds ratios and associated 95% confidence intervals (CI) for the collapsed measures of the substance-use variables shown above, using *year *as the predictor variable while controlling for gender differences. This yielded a measure of the odds of the average annual change in the likelihood of substance use for each variable.

## Results

### Daily smoking during last 30 days

**Figure 1:**
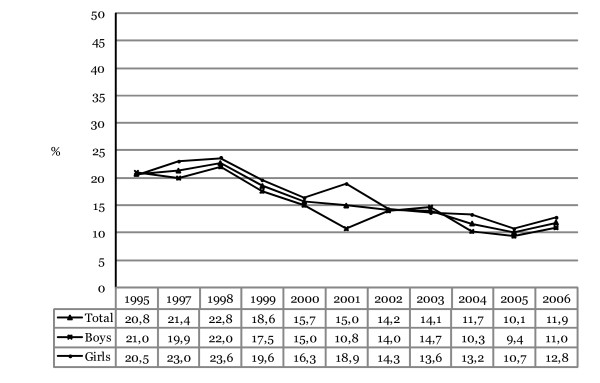
**Annual prevalence of daily smoking during the last 30 days among Icelandic 10^th^-grade students, 1995–2006**. The proportions shown for each year represent responses that have been collapsed into a dichotomized variable (0 = "Nothing or less than daily" and 1 = "Daily").

Figure 1 shows the prevalence of daily smoking during the last 30 days among adolescents in 10^th ^grade in Iceland from 1995 to 2006. The prevalence of smoking declined in total from a peaking 23% in 1998 to about 10% in 2005 but increased slightly again in 2006 with about 12% admitting to daily smoking. The total decrease in prevalence from 1995 (20.8%) to 2006 (11.9%) is about 43% (for trend analyses; *F*_linear _(1,11) = 700.36, p = .000; *F*_quadratic _(1,11) = 0.01, p = .921). Controlling for gender differences the odds of being a daily smoker decreased each year, on average, by 8.2% (OR = 0.918, 95% CI 0.910–0.925, Wald χ^2 ^(1) = 419.86, p = .000).

### Gender difference in daily smoking

As shown, the prevalence in daily smoking is on average greater among girls than boys. In 1995 only marginal differences were found between the sexes with 21.0% of boys admitting to daily smoking but 20.5% of girls smoking daily. For any other given year, with the exception of 2003, greater prevalence was discovered for girls with a peaking difference in 2001 when about 11% of boys admitted to daily smoking and close to 19% of girls. This large difference has decreased since then with 11.0% of boys admitting to daily smoking in 2006 and 12.8% of girls. The total decrease in prevalence is about 48% for boys (for trend analyses; *F*_linear _(1,10) = 306.64, p= .000; *F*_quadratic _(1,10) = 0.54, p= .464). The odds of being a daily smoker among boys decreased each year, on average, by 8.9% (OR = 0.911, 95% CI 0.901–0.922, Wald χ^2^(1) = 234.76, p = .000). The total decrease in prevalence is about 39% for girls (for trend analyses; *F*_linear _(1,10) = 254.58, p = .000; *F*_quadratic _(1,10) = 2.87, p = .090). The odds of being a daily smoker among girls decreased each year, on average, by 7.6% (OR = 0.924, 95% CI 0.914–0.934, Wald χ^2 ^(1) = 187.42, p = .000).

### Intoxication during last 30 days

**Figure 2:**
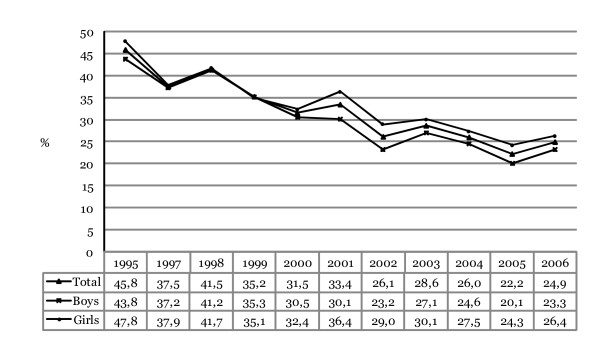
**Annual prevalence of intoxication during the last 30 days among Icelandic 10^th^-grade students, 1995–2006**. The proportions shown for each year represent responses that have been collapsed into a dichotomized variable (0 = "No" and 1 = "Yes, once or more often").

Figure 2 shows the prevalence of intoxication during the last 30 days among adolescents in 10^th ^grade in Iceland from 1995 to 2006. The prevalence of intoxication declined in total from a peaking 46% in 1995 to about 22% in 2005 but increased again in 2006 with about 25% admitting to being intoxicated once or more often during last 30 days. The total decrease in prevalence from 1995 (45.8%) to 2006 (24.9%) is about 46% (for trend analyses; *F*_linear _(1,11) = 956.40, p = .000; *F*_quadratic _(1,10) = 4.14, p = .042). Controlling for gender differences the odds of having been intoxicated decreased each year, on average, by 9.0% (OR = 0.910, 95% CI 0.904–0.916, Wald χ^2 ^(1) = 788.61, p = .000).

### Gender difference in prevalence of intoxication during last 30 days

As shown, the prevalence for intoxication is on average greater among girls than boys. In 1995 about 44% of boys admitted to having been intoxicated during the last 30 days and about 48% of girls admitted to intoxication. For any other given year, with the exception of 1999, greater prevalence was discovered for girls with a peaking difference in 2001 when about 30.1% of boys admitted to having been intoxicated during the last 30 days but more than 36% of girls. This large difference has decreased since then but remains about 3% in 2006 with 23.3% of boys admitting to intoxication and 26.4% of girls. The total decrease in prevalence from 1995 to 2006 is about 47% for boys (for trend analyses; *F*_linear _(1,10) = 370.68, p = .000; *F*_quadratic _(1,10) = 3.10, p = .078). The odds of intoxication during last 30 days among boys decreased each year, on average, by 9.7% (OR = 0.903, 95% CI 0.895–0.912, Wald χ^2^(1) = 448.16, p = .000). The total decrease in prevalence is about 45% for girls (for trend analyses; *F*_linear _(1,10) = 342.64, p = .000; *F*_quadratic _(1,11) = 4.50, p = .034). The odds of having been intoxicated during last 30 days among girls decreased each year, on average, by 8.3% (OR = 0.917, 95% CI 0.908–0.925, Wald χ^2^(1) = 344.47, p = .000).

### Hashish use in life time

**Figure 3:**
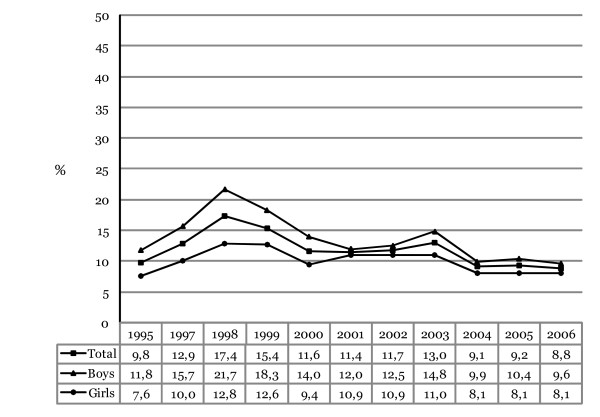
**Annual prevalence of those admitting to ever using hashish in their lifetime among Icelandic 10^th^-grade students, 1995–2006**. The proportions shown for each year represent responses that have been collapsed into a dichotomized variable (0 = "Never" and 1 = "Yes, at some point").

Figure 3 shows the prevalence of hashish use ever in life time among adolescents in 10^th ^grade in Iceland from 1995 to 2006. The prevalence of hashish use declined in total from a peaking 17.4% in 1998 to 8.8% in 2006. The total decrease in prevalence from 1995 (9.8%) to 2006 (8.8%) is only 10% but the decrease from 1998 to 2006 is 49% (for trend analyses; *F*_linear _(1,11) = 94.93, p = .000; *F*_quadratic_(1,11) = 95.14, p = .000). Controlling for gender differences the odds of having used hashish decreased each year, on average, by 3.7% (OR = 0.963, 95% CI 0.954–0.972, Wald χ^2 ^(1) = 64.58, p = .000).

### Gender difference in hashish use in life time

As shown, the prevalence for hashish use is on average greater among boys than girls. In 1995 about 12% of boys admitted to ever using hashish and 7.6% of girls reported hashish use ever in life time. For any given year greater prevalence was discovered for boys with a peaking difference in 1998 when about 22% of boys admitted to ever using hashish and about 13% of girls. This large difference has decreased a lot since then and was only marginal in 2006 with 9.6% of boys admitting to ever using hashish and 8.1% of girls. The total decrease in prevalence from 1995 to 2006 is about 19% for boys (for trend analyses; *F*_linear _(1,10) = 52.85, p = .000; *F*_quadratic _(1,10) = 43.18, p = .921). The odds of having ever used hashish among boys decreased each year, on average, by 5.1% (OR = 0.949, 95% CI 0.937–0.960, Wald χ^2 ^(1) = 71.18, p = .000). The change in prevalence between 1995 and 2006 among girls represents an increase of about 7% in total but the subsequent decrease between the peak in 1998 and 2005 is about 37% (for trend analyses; *F*_linear _(1,10) = 1.22, p = .269; *F*_quadratic_(1,10) = 33.23, p = .000). The odds of ever using hashish among girls decreased each year, on average, by 1.8% (OR = 0.982, 95% CI 0.968–0.996, Wald χ^2 ^(1) = 6.31, p = .012).

### Amphetamine use in life time

**Figure 4:**
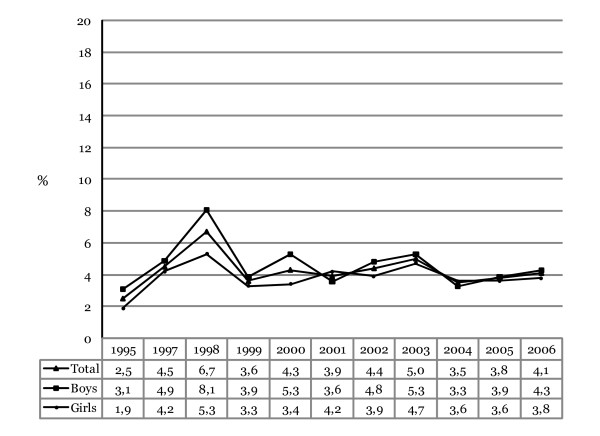
**Annual prevalence of those admitting to ever using amphetamines in their lifetime among Icelandic 10^th^-grade students, 1995–2006**. The proportions shown for each year represent responses that have been collapsed into a dichotomized variable (0 = "Never" and 1 = "Yes, at some point").

Figure 4 shows the prevalence of amphetamine use ever in life time among adolescents in 10^th ^grade in Iceland from 1995 to 2006. The prevalence of amphetamine use declined in total from a peaking 6.7% in 1998 to 3.5% in 2004 but increased slightly in 2005 and again in 2006 with 4.1% admitting to ever using amphetamine in their life time. The total change in prevalence from 1995 (2.5%) to 2006 (4.1%) represents an increase of 64%. However, the subsequent decrease from 1998 to 2006 is about 39% (for trend analyses; *F*_linear _(1,11) = 0.78, p = .781; *F*_quadratic _(1,11) = 29.61, p = .000). Controlling for gender differences the odds of ever using amphetamines did not change significantly on average each year (OR = 0.998, 95% CI 0.983–1.012, Wald χ^2 ^(1) = 0.11, p = .745).

### Gender difference in amphetamine use in life time

As shown, the prevalence in amphetamine use is on average greater among boys than girls. In 1995 just over 3% of boys admitted to ever using amphetamines and 1.9% of girls admitted to such use. For any other given year, with the exception of 2001 and 2003, greater prevalence was discovered for boys with a peaking difference in 1998 when 8.1% of boys admitted to ever using amphetamines in their life time and 5.3% of girls. This large difference has decreased since then with 4.3% of boys admitting to ever using amphetamines in 2006 and 3.8% of girls. The change in prevalence from 1995 to 2006 is an increase of about 39% for boys but the subsequent decrease from 1998 to 2006 is about 47% (for trend analyses; *F*_linear _(1,10) = 0.06, p = .808; *F*_quadratic_(1,10) = 7.31, p = .007). The odds of ever using amphetamines among boys did not change significantly on average each year (OR = 0.983, 95% CI 0.963–1.003, Wald χ^2 ^(1) = 2.80, p = .094). The total change in prevalence of amphetamine use from 1995 to 2006 among girls represents an increase of 100%. However the subsequent decrease from the peak in 1998 to 2006 is about 28% (for trend analyses; *F*_linear _(1,10) = 6.31, p = .012; *F*_quadratic_(1,10) = 8.68, p = .003). The odds of ever using amphetamines among girls did not change significantly on average each year (OR = 1.016, 95% CI 0.993–1.038, Wald χ^2 ^(1) = 1.85, p = .174).

## Discussion

This study found that substance use among Icelandic adolescents declined substantially over the 11-year period from 1995 to 2006. The observed declines are not likely to be due to secular change, but rather a response to the concerted substance-use prevention efforts that have been implemented in local communities during the course of the last decade in Iceland. In line with this notion two things are worth mention: First, the annual surveys revealed that in communities where local preventive work and collaborations with researchers had been the most active there was a substantially more reduction in adolescent substance use then in other areas. Second, within the areas were the downward trend in substance use was observed a clear pattern regarding the protective factors also repeatedly emerged. Thus, when identified protective factors such as time spent with parents, parental support and monitoring, and participation in organized sports and extracurricular activities increased continuously, substance use was decreasing.

Moreover, the Icelandic approach has highlighted the importance of addressing societal factors at multiple levels. It is worth noting that these changes in trends are both substantial and consistent. For example, from 1995 to 2006 the prevalence of daily smoking decreased about 43% and the prevalence of hashish use decreased about 49% from 1998 to 2006. Even more noteworthy is the reduction in intoxication during the last 30 days that decreased about 46% in the same time period. This is particularly interesting as the prevalence of alcohol use among this age group had not fluctuated but remained stable in Iceland for a long time [1]. The international ESPAD studies had revealed that Icelandic youths were more likely to become intoxicated once they consumed alcohol then many of their European peers [2]. A central focus in Iceland has therefore been to reduce alcohol intoxication among youth rather than just any alcohol use.

The decrease in prevalence of all the different substances has followed a similar path. This may indicate that the prevention efforts had a common core that influenced the use of all the substances simultaneously. It is also possible that alcohol may play a key role in substance use and smoking among adolescents and that the reduction in intoxication resulted directly in a reduction in the use of other substances. Nevertheless, although the Icelandic data documented the risk factors for substance use very well, it does not help us to explain why these fluctuations occur over time. It is clear that the trends in prevalence of substance use among young people have followed a path in Iceland different from many other European countries [2,3,20-22]. On the other hand, a similar trend has been documented in some of the Scandinavian countries, for example in Sweden and Norway, where substance use has decreased in recent years [2]. One exception to this is the prevalence of intoxication where Iceland shows a more consistent decrease than other European countries [2]. To gain a better understanding of these changes would require a systematic cross cultural comparison.

Overall, the downward trend in substance use follows a similar path for boys and girls. Interestingly, the prevalence of intoxication is consistently higher among girls than boys over the 11-year time period and they are slightly more likely to smoke daily. On the other hand, boys are slightly more likely to have used illegal substances throughout the time period but this gender difference has decreased substantially from 1998 to 2006.

While it is tempting to conclude that the comprehensive prevention effort launched in 1997 explains the reduction in substance use in Iceland it is, due to methodological limitations, impossible to claim with certainty that there is a statistical causal association between the decrease in substance use and the coordinated efforts to reduce the risk factors for adolescent substance use. However, the bulk of evidence from previous evaluations of the effects of the kind of substance-use prevention approach implemented in Iceland over the past decade suggests that focusing on the adolescent social environment have proven the most effective [3]. And furthermore, local trends in protective factors repeatedly indicate that this is the case. However, it complicates the picture that during the time period from 1997 to 2006 many preventive efforts were launched in various local communities and municipalities around Iceland. To monitor each and every one of them would not be feasible. It is, however, clear that an important shift occurred in Iceland in 1997 when changing the focus from the isolated individual to a more contextual understanding of the adolescent social world. Another important change was the emphasis placed on an evidence-based approach that linked research, policy, and practice. Today, ICSRA at Reykjavik University works closely with-, and has a long-term commitment to most municipalities around the country of Iceland while cooperating with the Ministry of Education. Nearly all the municipalities have developed a strategic plan which monitors adolescent substance use and risk behaviors, based on local data, collected annually by ICSRA in collaborations with the ministry of education and the local secondary schools. This has enabled the municipalities, its policy makers, and practitioners, to follow directly both local and national trends in substance use and subsequent risk behaviors, which then guides them to place emphasis on the most important matters each time. ICSRA researchers, local and national policy makers, and practitioners, are therefore engaged in continuous collaborations, which is updated on an annual bases.

The framing of the Icelandic substance-use prevention policy has drawn heavily from sociology and criminology, and combined elements from theories of learning, social capital, and social control. Also underlying this approach to substance-use prevention is a combination of ideas and findings from juvenile delinquency research and theories of social disorganization and anomie.

Some limitations to the study findings are worth noting. First, in reporting odds ratios for cigarette smoking we reveal data only for daily smoking, not *any *smoking, which has been a more widely reported indicator of tobacco use in the addiction literature [23], and second, the collection of data at a single point in time (March of each year) precludes capturing any seasonal variations that might exist in adolescent substance use in Iceland. Third, the nature of the Icelandic prevention approach prevents us from directly assessing if there is a causal link between the work being carried out by researchers, policy makers, and practitioners, both on local and national level in Iceland, and substance use.

From a policy perspective, the Icelandic experience has highlighted the importance of increasing opportunities for youth to participate in organized recreational work, such as sports and organized leisure activities. This participation fosters the opportunity to alter the atmosphere and culture within the adolescent peer group, which is the most important social force in the lives of adolescents that influences the likelihood of substance use [1,4,24]. Also, emphasizing parental support, monitoring, and time spent with their adolescent children has important implications for deterring substance use among adolescents [1,14,23]. Substance use prevention should, thus, aim at communicating these messages to parents through parental groups and schools and supporting parents through employment, flex-time, and other arrangements. Finally, the effect of cooperation between all relevant agents at the neighborhood level is of great importance. Encouraging and supporting parents, schools, local authorities, leisure-time workers, and others to work together on the local community level to promote adolescent health and well-being remains one of the greatest resources for boosting community social capital, which in turn appears to contribute significantly to the prevention of adolescent substance use [24,25]. Future studies that would compare the Icelandic experience with that of other countries in Europe and North America might therefore be of benefit in the battle against adolescent substance use.

## Conclusion

The prevalence of substance use among Icelandic 10^th ^graders declined substantially from 1995 to 2006. Proportions of adolescents who smoke cigarettes, had become intoxicated during the last 30 days, as well as those admitting to hashish use all decreased to a great deal during the period under study.

The Icelandic approach to adolescent substance use is not a single "project" in the conventional sense, with a given time frame and evaluation process from a certain beginning to a certain end; rather, it is a long-term strategy. Moreover, the Icelandic experience suggests that it is possible to address both the risk and protective factors for adolescent substance use without specifying a complex causal relationship between identified key factors and substance use. The findings of the current study also call for a closer look at the risk and protective factors for mental health problems among adolescents and their development over time. Icelandic studies, relying on annual surveys among 14 to 16 year old adolescents, have found that a number of protective factors within the family have evolved in a positive direction in the last few years. Both parental monitoring and adolescent's time spent with their parents has increased as well as the closeness of the parental network, known for being a protective factor against substance use. Future work would benefit from further research into the trends in risk and protective factors associated with substance use.

## Competing interests

The authors declare that they have no competing interests.

## Authors' contributions

IDS conceptualized the study, procured funding to support the original data collection and analyses, and contributed substantially to the writing and referencing, ÁLK assisted with conceptualizing the study, performed most of the data analyses, and contributed to the writing and referencing, TT contributed substantially to the original conceptualization of the study, writing, and edited multiple drafts of the manuscripts, JPA contributed substantially to the interpretation of the data analyses, original writing and referencing, and edited multiple drafts of the manuscript.

## References

[B1] Thorlindsson Th, Sigfusdottir ID, Bernburg JG, Halldorsson V (1998). Substance Use Among Young People [Vímuefnaneysla ungs fólks: Umhverfi og aðstæður] Reykjavík, Rannsóknarstofnun uppeldis- og menntamála;.

[B2] Hibell B, Barbro A, Bjarnason T, Ahlström S, Balakireva O, Kokkevi A, Morgan M (2003). The ESPAD Report 2003 Alcohol and Other Drug Use among Students in 26 European Countries.

[B3] Bauman A, Phongsavan P (1999). Epidemiology of substance use in adolescence: prevalence, trends and policy implications. Drug Alcohol Depend.

[B4] Thorlindsson Th (1989). Sport participation, smoking, and drug and alcohol use among Icelandic youth. Sociol Sport J.

[B5] Thorlindsson Th, Vilhjalmsson R (1991). Factors related to cigarette smoking and alcohol use among adolescents. Adolescence.

[B6] Huebner AJ, Betts SC (2002). Exploring the utility of social control theory for youth development–issues of attachment, involvement, and gender. Youth Soc.

[B7] Nagasawa R, Qian ZC, Wong P (2002). Social control theory as a theory of conformity: the case of Asian/Pacific drug and alcohol nonuse. Sociol Pers.

[B8] Costello BJ (2000). Techniques of neutralization and self-esteem: a critical test of social control and neutralization theory. Dev Beh.

[B9] Carver CS, Scheier MF (1982). Control-theory–a useful conceptual framework for personality, social, clinical, and health psychology. Psychol Bull.

[B10] Wiatrowski MD, Griswold DB, Roberts MK (1981). Social control theory and delinquency. Am Sociol Rev.

[B11] Dull RT (1983). An empirical examination of the anomie theory of drug use. J Drug Educ.

[B12] Lasky DI, Ziegenfuss JT (1979). Anomie and drug use in high school students. Int J Addictions.

[B13] Chamilin MB, Cochran JK (2007). An evaluation of the assumptions that underline institutional anomie theory. Theor Criminol.

[B14] Bjarnason T, Thorlindsson Th, Sigfusdottir ID, Welch MR (2005). Familial and religious influences on adolescent alcohol use: a multi-level study of students and school communities. Soc Forces.

[B15] Kristjansson AL, Sigfusdottir ID, Sigfusson J (2006). Young People in Iceland 2006 [Ungt Fólk 2006].

[B16] Hagstofa Íslands [Statistics Iceland] (2000). Statistical Yearbook of Iceland. Statistics of Iceland III, 82.

[B17] Bjarnason T (1995). Administration mode bias in a school survey on alcohol, tobacco and illicit drug use. Addiction.

[B18] Iversen GR, Norpoth H (1986). Analysis of variance.

[B19] Tabachnick BA, Fidell LS (2001). Using multivariate statistics.

[B20] Csemy L, Lejcková P, Sadílek P (2007). Substance use among Czech adolescents: an overview of trends in the international context. J Drug Iss.

[B21] Michaud P, Berchtold A, Eannin A, Chossin I, Suris JC (2006). Secular trends in legal and illegal substance use among 16–20-year-old adolescents in Switzerland. Swiss Med Wkly.

[B22] Csemy L, Kubicka L, Nociar A (2002). Drug scene in the Czech Republic and Slovakia during the period of transformation. Eur Addict Res.

[B23] Kristjansson AL, Sigfusdóttir ID, Allegrante JP, Helgason ÁR (2008). Social correlates of cigarette smoking among Icelandic adolescents: a population-based cross-sectional study. BMC Public Health.

[B24] Thorlindsson Th, Bjarnason T, Sigfusdottir ID (2007). Individual and community processes of social closure: a study of adolescent academic achievement and alcohol use. Acta Sociol.

[B25] Lundborg P (2005). Social capital and substance use among Swedish adolescents-an explorative study. Soc Sci Med.

